# Ultrasound point shear wave elastography of the pancreas: comparison of patients with type 1 diabetes and healthy volunteers – results from a pilot study

**DOI:** 10.1186/s12880-018-0295-z

**Published:** 2018-12-13

**Authors:** Sophie Püttmann, Janina Koch, Jochen Paul Steinacker, Stefan Andreas Schmidt, Thomas Seufferlein, Wolfgang Kratzer, Julian Schmidberger, Burkhard Manfras

**Affiliations:** 1 Department of Internal Medicine I, University Hospital Ulm, Albert-Einstein-Allee 23, 89081 Ulm, Germany; 2 Department of Diagnostic and Interventional Radiology, University Hospital Ulm, Albert-Einstein-Allee 23, 89081 Ulm, Germany; 3 Medicover Medical Centre, Münsterplatz 6, 89073 Ulm, Germany

**Keywords:** Type 1 diabetes, Ultrasound p-shear wave elastography, Healthy volunteers, Pancreas

## Abstract

**Background:**

The aims of this study were to establish shear wave elastography of the pancreas by comparing measurements in patients with type 1 diabetes (T1D) and healthy volunteers and to consider whether this method could contribute to the screening or prevention of T1D.

**Methods:**

This pilot study included 15 patients with T1D (10 men, 5 women) and 15 healthy volunteers (10 men, 5 women) as controls. Measurements were performed with a Siemens Acuson S3000 (Siemens Healthcare, Erlangen, Germany) using a 6C1 convex transducer and the Virtual Touch™ tissue quantification (VTQ) method.

**Results:**

The mean shear wave velocity of the head of the pancreas was 1.0 ± 0.2 m/s (median: 1.1 m/s) for the study group and likewise 1.0 ± 0.2 m/s (median: 0.9 m/s) for the control group. Velocities of 1.2 ± 0.2 m/s (median: 1.2 m/s) were measured in the body of the pancreas in both groups. There was a significant difference between the values obtained in the tail of the pancreas: patients 1.1 ± 0.1 m/s (median: 1.0 m/s) versus controls 0.9 ± 0.1 m/s (median: 0.8 m/s) (*p* = 0.0474). The mean value in the whole pancreas of the study group was not significantly above that of the control group: 1.1 ± 0.1 m/s (median: 1.0 m/s) versus 1.0 ± 0.1 m/s (median: 1.0 m/s) (*p* = 0.2453).

**Conclusions:**

Sonoelastography of the pancreas revealed no overall difference between patients with T1D and healthy volunteers. Patients with T1D showed higher values only in the tail segment. Future studies need to determine whether specific regional differences can be found in a larger study population.

## Background

Type 1 diabetes (T1D) is an autoimmune disease in which insulin-producing beta cells in the pancreas are destroyed. Comparative international figures show an annual increase of 3–4% in the global incidence rates of T1D [1]. Since there is a strong genetic contribution to T1D, further screening modalities could be of interest for patients at genetic risk [2]. Shear wave elastography presents a method for determining the elasticity of the target tissue, using ultrasound to determine the velocity of shear waves [3]. The procedure was originally developed as a non-invasive investigation for the early diagnosis and treatment of liver fibrosis [4]. D’Onofrio et al. and Kawada et al. described ductal adenocarcinoma as stiffened tissue with an increased shear wave velocity (Vs) due to the fibrotic process [3, 4]. Studies have also shown an increase in the Vs in patients with chronic pancreatitis [5]. Recent studies on type 2 diabetes (T2D) have demonstrated that elastographic examination of the pancreas shows an increased Vs in affected patients compared with healthy subjects [6]. Concerning type 1 diabetes, a recent study on children and adolescents did not show any increased values in those with T1D. However, that study looked only at the body of the pancreas [7].

It is assumed that pathological mechanisms associated with fibrotic change cause the inflamed tissue to increase stiffness [3–5]. As inflammation in the islets of Langerhans (insulitis) and fibrosis also occur in T1D [8–10], questions arise as to whether the inflammatory process and the associated alteration in tissue consistency can be measured by shear wave elastography and whether changes can be detected even in the prediabetic phase, when an insulitic process is already taking place. As the results of the various elastographic studies show a certain lack of agreement regarding both examination precision and current clinical relevance [3, 5], this procedure has still to be established for the diagnostic investigation of pancreatic disease.

Although a growing understanding of the pancreatic pathology is allowing the development of novel immune intervention strategies to alter the course of insulitis, additional non-invasive diagnostic tools are still desirable for the early diagnosis of T1D and monitoring the course of disease [2].

The aims of this pilot study were therefore to establish shear wave elastography in the three anatomical regions of the pancreas (head, body, and tail) and evaluate possible alterations in stiffness due to the pathology of T1D by comparing elastographic measurements in patients with T1D and healthy controls.

## Methods

### Patients and healthy volunteers

The study population comprised 15 patients with T1D and 15 healthy volunteers as controls. Baseline characteristics are summarised in Table 1. We recruited the patients with type 1 diabetes from the hospital diabetes outpatient clinic and an endocrinology practice. Patients had been diagnosed previously with T1D in accordance with the guidelines and irrespective of the study [11]. The control group consisted of healthy volunteers selected to match the individual patients for age and sex. The following exclusion criteria were applied to the whole study population:

- disease or surgery of the pancreas (acute or chronic pancreatitis, pancreatic cancer, partial or complete resection of the pancreas)
- disease of the liver or biliary tract (hepatitis, primary sclerosing cholangitis (PSC), primary biliary cirrhosis (PBC), alcoholic steatohepatitis (ASH), non-alcoholic steatohepatitis (NASH), cirrhosis of the liver, and portal hypertension)
- BMI > 30 kg/m^2^
- fasting for < 6 h
- alcohol abuse (> 20 g alcohol for women, > 40 g alcohol for men)
- weight fluctuation +/− 10 kg in the last 3 months
- pregnancy.

**Table 1:** Overview of the study population

	Mean ± SD median (min-max) IQR			
	Whole population	Patients with type 1 diabetes	Healthy volunteers	*p*-value
Sex				
Male	20 (66.7%)	10 (66.7%)	10 (66.7%)	1.0
Female	10 (33.3%)	5 (33.3%)	5 (33.3%)	
Age [years]	31.8 ± 9.6	32.1 ± 9.5	31.4 ± 10.1	0.9006
	30 (20.0–54.0)	30.0 (20.0–50.0)	28.0 (21.0–54.0)	
	17,0	17,0	17,0	
BMI [kg/m^2^]	23.7 ± 2.7	24.3 ± 3.0	23.3 ± 2.4	0.3947
	22.9 (19.9–29.4)	24.8 (19.9–29.4)	22.3 (20.8–28.9)	
	3,7	5,1	3,0	
Duration of diabetes [months]		45.7 ± 42.3		
		25.0 (5.0–125.0)		
		61,0		
HbA_1c_ [%]		6.7 ± 0.9	5.2 ± 0.2	< 0.0001
		7.0 (5.4–8.0)	5.2 (4.8–5.6)	
		1,5	0,3	
Fasting glucose [mg/dl]		111.2 ± 37.0	90.0 ± 7.8	0.0777
		102.0 (66.0–191.0)	89.0 (73.0–104.0)	
		51,0	11,0	
C peptide^a^ [μg/l]		0.9 ± 0.7	1.6 ± 0.5	0.0082
		0.7 (0.1–2.0)	1.6 (0.8–2.8)	
		1,1	0,4	
Basal insulin (IU/d]		13.9 ± 7.4		
		12.0 (2.0–30.0)		
		11,0		

*SD* Standard deviation, *min* Minimum, *max* Maximum, *IU* International unit, *level of significance P* < 0.05, *IQR* Inter quartile range

^a^C peptide was not measured in one female patient

Endocrine diseases were exclusion criteria in healthy volunteers but not in patients, since T1D is not uncommonly associated with other endocrine disorders.

Further exclusion criteria for the healthy volunteers were an HbA_1c_ of 5.7–6.4% (prediabetic range) or > 6.4%, as well as positive antibodies (IAA, IA2, GAD65). These parameters were measured in a venous blood sample from each participant. Data on the medical history were collected with a standardised questionnaire and we obtained additional information about the onset of the disease, duration, and treatment regimen from the patients. The study was conducted in conformity with the principles of the Helsinki Declaration and Good Clinical Practice and was approved by the local Ethics Committee (No. 331–15, 1 September 2015). All participants enrolled in the study gave their written informed consent.

Twenty-one patients with T1D and 17 healthy volunteers initially participated in our elastography study. Six patients and two healthy volunteers were subsequently excluded. One patient had no islet-cell autoantibodies. This patient and one other had a BMI over the limit of 30 kg/m^2^. Two male patients were excluded because of high alcohol consumption > 40 g/d. A marked fluctuation in weight over the past 3 months led to the exclusion of two more patients. One patient had lost more than 10 kg in weight, while the other had gained more than 10 kg during this period.

Blood tests in one of the healthy volunteers revealed diabetes antibodies (GAD65 and IA2) leading to exclusion from the control group. Another healthy volunteer was excluded because of a fasting period less than 6 h.

### Elastography

**Fig. 1:**
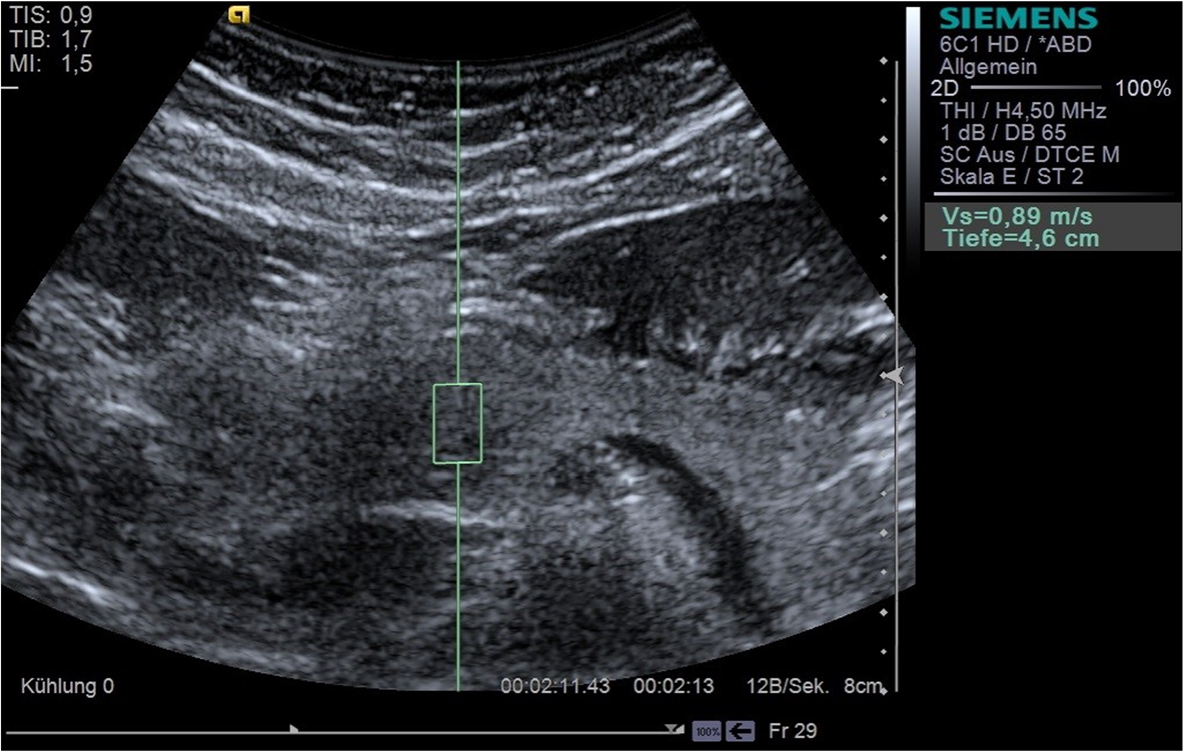
Measurement of the shear wave velocity (Vs) of the head of the pancreas with VTQ

**Fig. 2:**
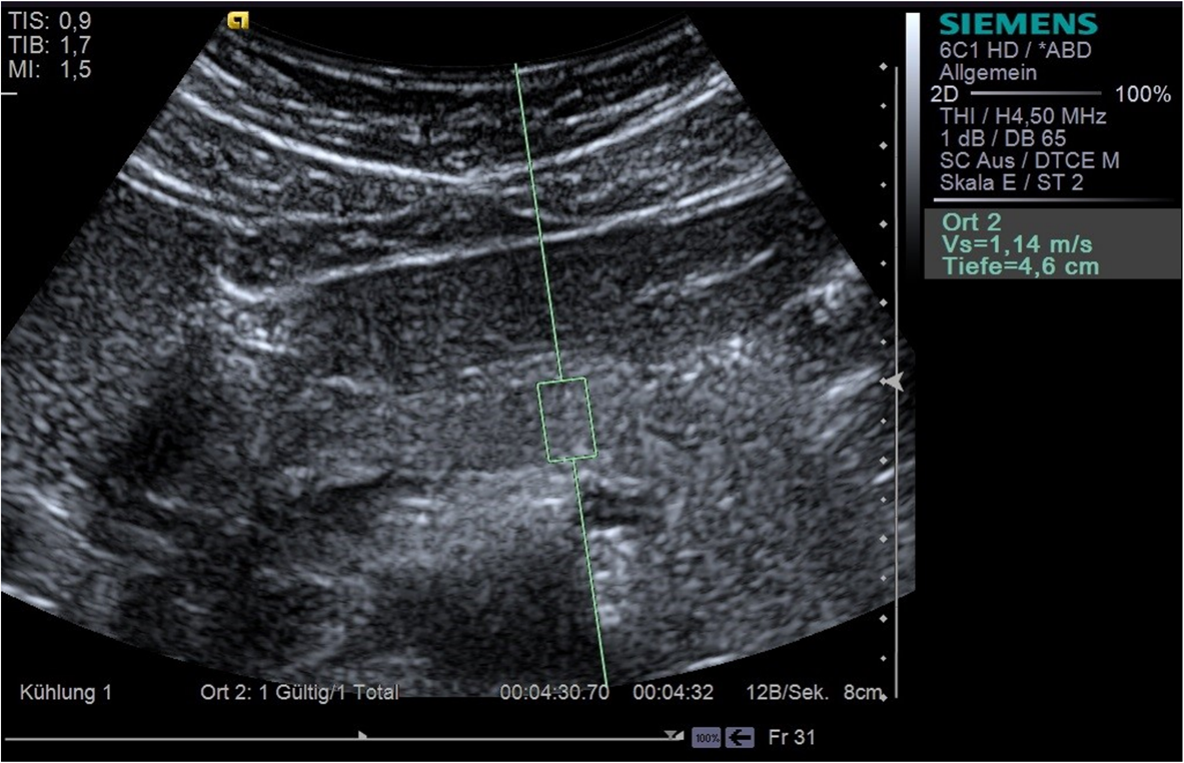
Measurement of the shear wave velocity (Vs) of the body of the pancreas with VTQ

**Fig. 3:**
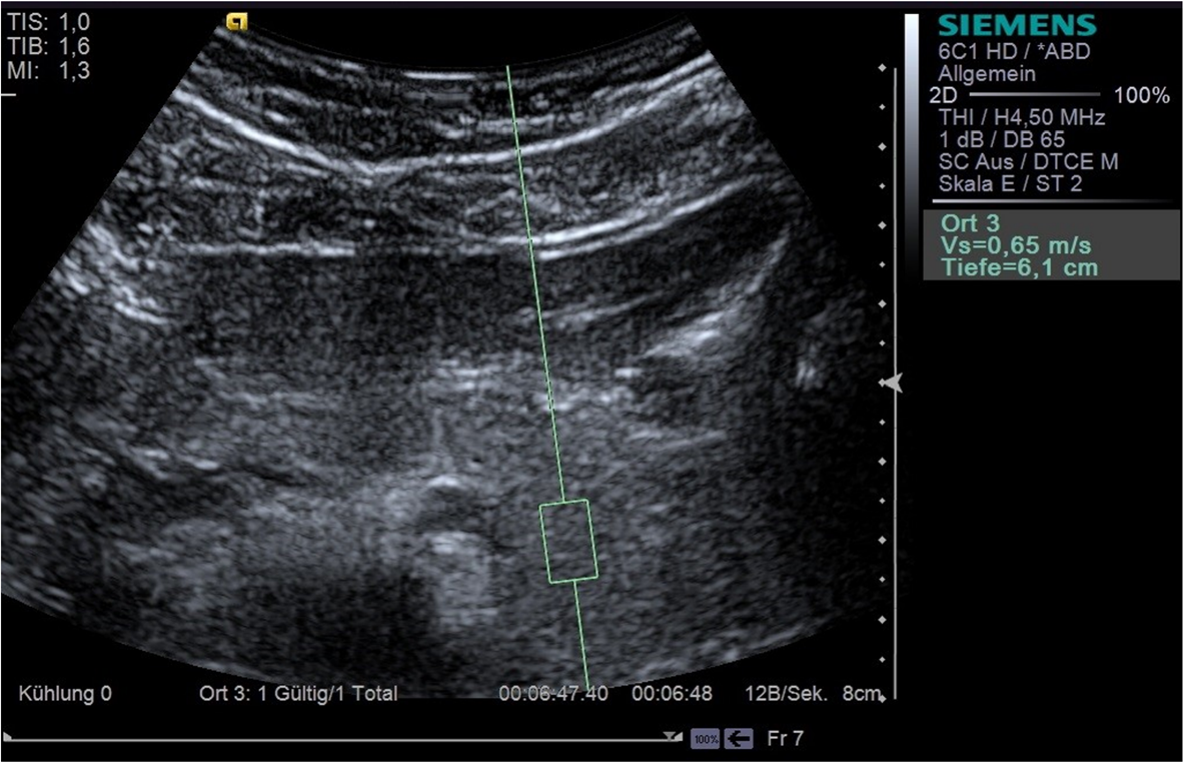
Measurement of the shear wave velocity (Vs) of the tail of the pancreas with VTQ

All p-shear wave elastographic measurements were carried out with Virtual Touch™ Quantification (VTQ) on a Siemens Acuson S3000 using a 6C1 convex transducer (Figs. 1, 2 and 3). VTQ is based on the technique of acoustic radiation force impulse (ARFI) imaging, using ultrasound waves to determine the tissue stiffness quantitatively and calculate the numerical Vs. At the start of each investigation, the pancreas was demonstrated in B-mode and the upper abdomen assessed to rule out any hepatic or cholestatic disease. In this study, a 10 × 5 mm region of interest (ROI) was selected for each pancreatic segment (head, body, and tail) and at least five elastographic measurements taken in each case. The confluence of the splenic and superior mesenteric veins was taken to mark the boundary between head and body. The tail of the pancreas was identified as the structure anterior to the left kidney, extending to the hilum of the spleen. It was particularly important to ensure that no blood vessels were located within the ROI, since pulsations (including those from the aorta) can interfere with ARFI [12]. Participants were positioned supine; they were asked to exhale completely and hold their breath during each Vs measurement in order to reduce motion artefacts as much as possible. The mean and standard deviation were calculated for each pancreatic segment, and the median value also given in units of m/s. A single examiner, who was not blinded with respect to the diagnosis of diabetes, carried out all the measurements. The Vs measurements were also checked for correlation with the duration of diabetes and the BMI of both patients and healthy volunteers.

### Statistical analysis

We used SAS 9.2 software (SAS Institute Inc., Cary, North Carolina, USA) for the statistical analysis. The mean, standard deviation, median, and the range (minimum-maximum) were calculated as continuous variables in each case. Discrete variables were given with absolute and relative frequencies. We used the Wilcoxon rank sum test to show any differences in continuous variables between two groups (e.g. patients and healthy volunteers or men and women) and chose the Wilcoxon signed rank test to compare two continuous variables. Correlation of parameters was calculated with Spearman’s rank correlation coefficient. For all the statistical analyses, a *p*-value of less than 0.05 in two-tailed tests was considered to be significant. Gpower Version 3.1. was used for statistical power analysis and sample size analysis. Assuming a power of greater than 80% (1-β), the analysis yielded a total sample size of *n* ≥ 30 for the statistical methods and the pilot study.

## Results

Tissue elasticity was measured in the three pancreatic segments: head, body and tail. The mean overall velocity measured in the control group was 1.0 ± 0.1 m/s. The corresponding figure in the study group was 1.1 ± 0.1, showing no significant difference (*p* = 0.2453).

The shear wave velocity of the tail differed significantly between patients with T1D and healthy volunteers indicating a higher stiffness in T1D (*p* = 0.0474) (Table 2). In contrast, the Vs in the head and body was not significantly different between the two groups. With respect to the elastographic measurements, it was also of interest to establish the segment of the organ showing the highest and lowest velocity in each group. Comparing the means, we found the highest velocity in the body of the pancreas in both groups (1.2 ± 0.2).

**Table 2:** Elastographic measurements in the head, body and tail of the pancreas of patients with type 1 diabetes and healthy volunteers

Vs in [m/s]	Mean ± SD median (min-max) IQR		
	Patients with type 1 diabetes	Healthy volunteers	*p*-value
Site 1 = head	1.0 ± 0.2^a^	1.0 ± 0.2	0.4193
	1.1 (0.7–1.5)	0.9 (0.6–1.3)	
	0,3	0,2	
Site 2 = body	1.2 ± 0.2	1.2 ± 0.2	0.9834
	1.2 (0.9–1.8)	1.2 (0.9–1.6)	
	0,18	0,4	
Site 3 = tail	1.0 ± 0.2^b^	0.9 ± 0.1	0.0474
	1.0 (0.8–1.3)	0.8 (0.7–1.2)	
	0,04	0,2	
Mean (1–3)	1.1 ± 0.1	1.0 ± 0.1	0.2453
	1.0 (0.9–1.4)	1.0 (0.8–1.2)	
	0,2	0,2	

Vs Shear wave velocity, *SD* Standard deviation, *min* Minimum, *max* Maximum, *IQR* Inter quartile range, *level of significance P* < 0.05

^a^1 Missing from 1 patient because a penetration depth > 8 cm not possible, only site 2 could be measured in this patient

^b^Missing from 2 patients

In the control group of healthy volunteers, we found a positive correlation with age for the individual pancreatic segments of the head and body, as well as for the whole pancreas (head: *p* = 0.0009, *R* = 0.76308; body: *p* = 0.0022, *R* = 0.72589; whole pancreas: *p* = 0.0012, *R* = 0.75430) but established no such relationships in the patient group. There was no association between Vs and duration of diabetes in the study group for either of the individual pancreatic segments or the whole organ (*p* = 0.7534) nor was there any correlation between BMI and Vs in either group (patients: *p* = 0.7129; controls: *p* = 0.5402). The position of the pancreas in the abdomen meant that the maximum ultrasound penetration depth of 8 cm was insufficient in one patient and made it impossible to take any measurements in the deeper-lying segments. Large quantities of intestinal air can also make it more difficult or even impossible to measure the pancreas. For this reason, the tail segment could not be measured in two of our patients.

## Discussion

Both ultrasound strain elastography and ultrasound shear wave elastography appear to play an increasing role in the diagnostic investigation of pancreatic disease [13, 14]. Despite the increasing importance of elastographic procedures in the diagnosis of pancreatic disease, we are aware of only a few studies on shear wave elastography of the pancreas in patients with diabetes [15, 16]. In our pilot study, we intended to demonstrate the feasibility of measuring all three anatomical parts of the pancreas and determine measurement limitations with respect to patient characteristics and examination conditions.

Our results (Table 2) show a significant difference between patients and healthy volunteers in the tail of the pancreas (*p* = 0.0474). The mean value for all segments showed only a trend towards a higher Vs in the patient group.

In a recent study, Saglam et al. did not find any significant differences between children and adolescents with T1D and healthy subjects, but the measurements were performed only on the body of the pancreas. This finding agrees with our results for that region. They reported Vs of 1.09 ± 0.22 m/s for the body of the pancreas in healthy control subjects and 0.99 ± 0.25 m/s for patients with type 1 diabetes – values slightly lower than we found in our patients [7].

Chronic inflammatory processes lead to fibrotic remodelling consistent with non-physiological wound healing not only in the islets of Langerhans (insulitis) but also in the exocrine tissue of the pancreas [17–19]. Pancreatic fibrosis particularly affects patients with longstanding disease [10, 20]. In our study, the longest duration of disease was about 10 years.

The higher velocities detected may not be due exclusively to fibrosis, as other histological changes may impact the physical properties of the tissue. But fibrotic changes are known to result from type 1 diabetes [17, 21, 22].

One possible explanation for our significant results from the tail could be a greater accumulation of inflammatory CD8+ T cells in this organ segment during insulitis. We can assume that these cells are not only responsible for considerable destruction of the beta cells but are also to be found in greater numbers in the exocrine tissue [19]. In the context of recurrent inflammation of the islets of Langerhans in T1D, Rajput et al. described a higher concentration of beta cells in the tail of the pancreas [10]. This would suggest that patients with T1D have stiffer tissue in the tail of the pancreas, giving higher Vs values in this segment.

Harada et al. used shear wave elastography with ultrasound to examine the body of the pancreas preoperatively in patients about to undergo pancreatic resection. They showed a strong correlation of Vs to the severity of pathological fibrosis in the pancreatic tissue [15].

Stumpf et al. found normal values for the head of the pancreas to be 1.44 ± 0.39 m/s for women and 1.19 ± 0.29 m/s for men. Values for the body were 1.49 ± 0.37 m/s for women and 1.26 ± 0.30 m/s for men, while the corresponding figures for the tail were 1.29 ± 0.36 m/s and 1.05 ± 0.30 m/s, respectively [14]. The values given by Harada et al., with 1.35 m/s for the body of the pancreas, are to a large extent in agreement with the values given by Stumpf et al. and our measurements [15]. Their results for male participants agree extremely well with our results. As the percentage of women in our healthy control group was only 30%, this may explain why the values in the literature for the two sexes combined are slightly higher than our results. Yashima et al. found mean values for head, body and tail of 1.23 m/s, 1.3 m/s and 1.24 m/s in healthy volunteers and of 1.65 m/s, 2.09 m/s and 1.68 m/s in patients with chronic pancreatitis [5]. Xie et al. reported mean values for the pancreatic head and body of 1.18 m/s and 1.21 m/s in healthy controls [23]. The data from Goertz et al. are in accordance with these measurements, with a mean of 1.2 m/s. The average for patients with chronic pancreatitis in that study was 2.21 m/s [24]. Mateen et al. reported results of 1.28 m/s, 1.25 m/s, and 3.28 m/s for healthy controls, patients with chronic pancreatitis, and patients with acute pancreatitis, respectively [25]. Llamoza-Torres et al. obtained figures of 1.27 m/s for healthy volunteers and 1.57 m/s for patients with chronic pancreatitis [26]. Further studies evaluate ARFI as a suitable and promising procedure for the non-invasive diagnostic investigation of chronic pancreatitis and other pancreatic conditions, both benign and malignant, such as cystic lesions and carcinomas [12, 26–29]. In the literature, the highest Vs was recorded in the body of the pancreas whenever the individual pancreatic segments were measured. Our results confirm these findings, as the Vs of the body of the pancreas was significantly higher in both groups.

In the healthy volunteers, we found a very strong relationship between the Vs and the age of the participant, confirming the observation of Stumpf et al. [14]. Nevertheless, in contrast to their study, we did not find any correlation between Vs and BMI. Nor did we determine a correlation between the Vs and the duration of diabetes (*p* = 0.7534), a finding which agrees with the results published by Saglam et al. [7].

Although our pilot study comprised only 30 participants and is therefore of limited statistical power, our results compare well with previously published data on pancreas elastography. Since one very experienced examiner carried out all the measurements, we eliminated any bias from different examiners. However, the ultrasound images were not checked by a second reader, which is a definite limitation of the study. Our examiner was not blinded to the participant being a healthy volunteer or a patient. In the present study, our findings were statistically significant in the tail of the pancreas: this part of the organ is the most difficult to examine with imaging techniques and might therefore also be the most inaccurate. The precision of Vs measurement by shear wave elastography may be affected by vascular pulsation and the maximum depth of shear wave detection may be a further limitation, especially in obese patients [12].

For such a detailed analysis of changes in pancreatic morphology in T1D, factors that would limit the measurement precision, as well as variations in the examination conditions, led to individuals being excluded from the study population. Furthermore, the average duration of diabetic disease in our patients was 45 months, which is not a very long time in the course of T1D. Results could be different in patients who have had the disease for a longer period.

## Conclusion

In conclusion, we have presented the first elastographic measurements of all pancreatic segments in patients with T1D. We found no overall difference between patients and healthy volunteers. There is only a trend indicating that chronic inflammatory processes in T1D may lead to stiffer tissue in the tail of the pancreas. Elastography is a non-invasive, reliable, and rapidly available procedure offering additional information in the diagnostic investigation of pancreatic disease. Especially for patients at risk of T1D, it might represent a promising imaging modality for screening purposes. Additional studies need to clarify whether a higher Vs is associated with T1D-specific pancreatic changes and whether this finding is related to the duration of diabetes.

## Abbreviations

ARFI: Acoustic radiation force impulse
ROI: Region of interest
T1D: Type 1 diabetes mellitus
Vs: Shear wave velocity
VTQ: Virtual Touch™ Quantification
